# Amodal Completion of a Target Template Enhances Attentional Guidance in Visual Search

**DOI:** 10.1177/2041669518796240

**Published:** 2018-08-30

**Authors:** Siyi Chen, Lucas Schnabl, Hermann J. Müller, Markus Conci

**Affiliations:** Ludwig-Maximilians-Universität München, Germany

**Keywords:** amodal completion, global completion, local completion, visual search, target template

## Abstract

When searching for a target object in cluttered environments, our visual system appears to complete missing parts of occluded objects—a mechanism known as “amodal completion.” This study investigated how different variants of completion influence visual search for an occluded target object. In two experiments, participants searched for a target among distractors in displays that either presented composite objects (notched shapes abutting an occluding square) or corresponding simple objects. The results showed enhanced search performance when composite objects were interpreted in terms of a globally completed whole. This search benefit for global completions was found to be dependent on the availability of a coherent, informative simple-object context. Overall, these findings suggest that attentional guidance in visual search may be based on a target “template” that represents a globally completed image of the occluded (target) object in accordance with prior experience.

## Introduction

**Figure 1.**
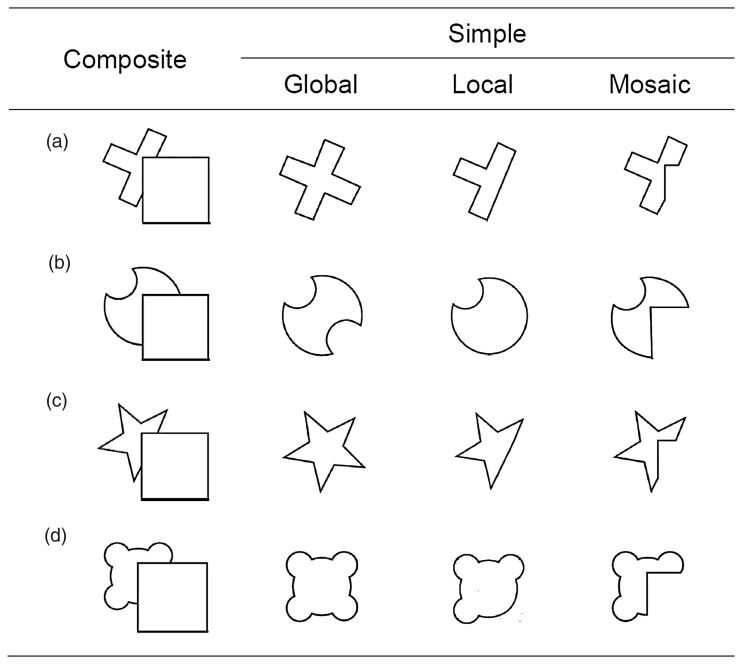
Illustration of the experimental stimuli with their respective composite and simple versions (depicting global or local completion variants, or a corresponding mosaic interpretation).

A ubiquitous obstacle that the visual system encounters in object perception is occlusion. For instance, parts of the objects in our typically complex, cluttered environments may lack a visual stimulus correlate, because they are partly occluded by other structures—which may make it hard to find some searched-for but partially occluded target object. To overcome this limitation and establish continuity, amodal completion has been described as a mechanism that fills in missing perceptual information (Michotte, Thinès, & Crabbé, 1964/1991). Object integration across occlusions is usually ambiguous and may, for instance, involve local or global completion (R. J. Van Lier, Leeuwenberg, & Van der Helm, 1995). For example, as illustrated in Figure 1, a local completion constitutes a smooth continuation of the visible contours of the background shape behind the occluding square (Kellman & Shipley, 1991). Conversely, global completion emphasizes global relations of the occluded object, in particular symmetry (Buffart, Leeuwenbert, & Restle, 1981). Another alternative might represent an occluded figure in terms of a “mosaic,” that is, without amodal completion.

Previous studies investigating amodal completion using visual search paradigms demonstrated that, when observers are provided with sufficient time for encoding, detection of a partially occluded target item relies on a completed object representation (He & Nakayama, 1992; Rauschenberger & Yantis, 2001; Rensink & Enns, 1998). For instance, search for a notched target disk abutting an occluding square, in an array of complete distractor disks and squares, turned out to be relatively inefficient—suggesting that the notched target is represented as a complete object, that is, as being rather similar to the distractors (Rauschenberger & Yantis, 2001; Rensink & Enns, 1998). Other studies showed that distractors in a search display bias the interpretation of a given composite target figure (Rauschenberger, Peterson, Mosca, & Bruno, 2004), indicating that a given interpretation of an object depends on the context within which it is presented. However, in these studies, object completion usually results in an unambiguous representation (e.g., a circle completed behind a square), whereas in natural scenes, occluded objects may be ambiguous and local and global processes may lead to qualitatively different interpretations of the very same visual input (R. J. Van Lier, Van der Helm, & Leeuwenberg, 1995). To address this issue of object ambiguity, this study investigated how different variants of completion influence visual search for a partially occluded target.

Of note in this context, recent work has shown that visual working memory tends to represent occluded objects in terms of globally completed wholes, rather than local completions (Chen, Müller, & Conci, 2016; Chen, Töllner, Müller, & Conci, 2018; for stimuli, see Figure 1). A benefit of global over local completions was also reported in studies that used an implicit primed matching task (Sekuler, Palmer, & Flynn, 1994; R. Van Lier, 1999). Given this pattern of evidence, one might predict that visual search for an occluded target among (occluded) distractors is likewise governed by global, but not local, completions. This study was designed to test this hypothesis, by investigating how various types of completion impact visual search performance. Observers were required to detect a target among distractors in displays that either presented composite objects (notched shapes abutting an occluding square) or corresponding simple object variants that would correspond to global, local, or mosaic interpretations of the composite objects (see Figure 1). To effectively enforce a given interpretation of the composite objects, observers were provided with a consistent context of simple-object displays among ambiguous composite object displays (Chen et al., 2016)—so as to examine whether the various completion types would differentially affect visual search performance.

## Experiment 1

### Method

#### Participants

Sixteen volunteers (six males, two left-handed) with normal or corrected-to-normal vision participated in Experiment 1 (mean age = 20.94 years), either for payment of €8.00 per hour or for course credits. All observers were naive as to the purpose of the study and provided written informed consent. The experimental procedure was approved by the ethics committee of the Department of Psychology, Ludwig-Maximilians-University, Munich.

#### Apparatus and stimuli

**Figure 2.**
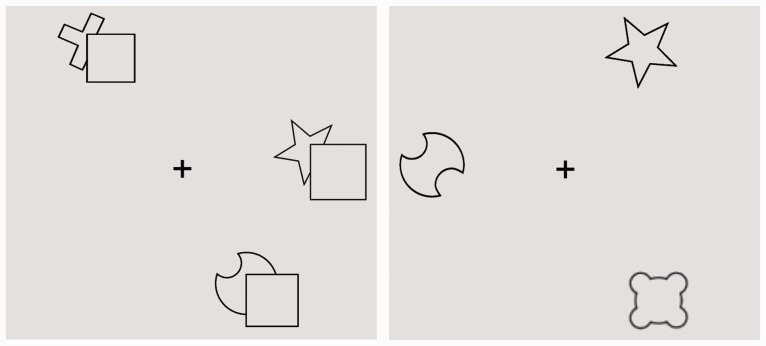
Left: Example three-item search display with composite objects (target-present trial with the to-be detected target cross in the top-left corner of the display). Right: Example of a three-item display presenting simple, global completion objects (target-absent trial without a cross-shaped target).

The experiment was controlled by a Windows 7 computer, running Matlab and Psychophysics Toolbox (Brainard, 1997). The stimuli were black line drawings (0.2 cd/m^2^) against a gray background (178 cd/m^2^), presented on a 17-in. CRT monitor (1,024 × 768 pixel screen resolution, 85 Hz refresh rate). The stimulus set consisted of composite and simple objects (see Figure 1), which was adapted from previous studies (Chen et al., 2016; see also Plomp & Van Leeuwen, 2006; Sekuler et al., 1994). Each composite figure included a square (2.1° × 2.1°) with a second shape positioned partly occluded next to the square (Figure 1, composite). Every simple figure was presented in three variants, corresponding to three possible alternative interpretations of the composite object: global completion, local completion, and mosaic (see Figure 1, simple). Global completions presented a symmetrical shape interpretation of the occluded object, whereas a local completion was based on the smooth continuation of the visible parts of the occluded shape. A mosaic figure simply presented a 2D cutout outline shape identical to the visible part of the partly occluded figure. The widest aspect of each simple object touched the borders of a circular region with a radius of 2.4° of visual angle. For each search display, three, five, or seven distinct objects from the same completion condition (with a given shape appearing up to three times in the same display) were presented randomly at nine positions within a circular region subtending 12.4° of visual angle. Figure 2 shows two example search displays.

#### Procedure and design

Each trial started with the presentation of a central fixation cross for 500 ms. Next, participants were presented with a search display of simple or composite objects until they responded. Their task was to search for a cross-shaped target (i.e., one of the items displayed in Figure 1(a)), and to indicate, by pressing the left/right mouse key, whether the target was present/absent, respectively. The target was always a variant of the cross-shaped stimulus (Figure 1(a)), with the other three object types serving as the distractor shapes (Figure 1(b)–(d)). Observers were asked to respond as quickly and as accurately as possible. In case of an erroneous response, feedback was provided by presenting an “alerting” sign (“–”) for 1,000 ms at the center of the screen. Trials were separated from each other by an interval of 500 ms.

The experiment was subdivided into three parts, with each part consistently displaying one type of simple object shape representations—that is, global, local, or mosaic—in the search displays. Thus, in each part, half of the trials presented displays consisting of one particular type of simple objects, which were presented randomly intermixed with the other half of trials that displayed (ambiguous) search layouts of composite objects (see Figure 2). Thereby, we were able to enforce a corresponding interpretation of the composite (occluded) objects within a given experimental part (Chen et al., 2016). The order of presentation of the three parts was randomized. Within each part, the different configuration (simple and composite), set size (three, five, or seven items) and target (present and absent) conditions were presented in randomized order across trials. There was one initial block of 12 practice trials, followed by 372 trials per part (yielding 1,116 experimental trials in total), with short breaks after every 124 trials in each part.

### Results

The analysis of the error rates and reaction times (RTs) focused on target-present trials, following previous search studies investigating amodal completion (Rauschenberger & Yantis, 2001; Rauschenberger et al., 2004). Target-present trials were primarily analyzed because they provide a match between the memory representations of the to-be-searched-for target with one of the objects present in the display. On target-absent trials, the decision when to terminate search when no target is found may add substantial variance to the response times (Chun & Wolfe, 1996), which is why these trials were discarded from the analyses presented here.

#### Errors

The mean overall error rate was 3.2%. Errors were examined by means of a repeated-measures analysis of variance (ANOVA), with the factors object (simple and composite), interpretation (global, local, or mosaic), and set size (three, five, and seven). This ANOVA revealed main effects of object, *F*(1, 15) = 16.41, *p* = .001, $\eta_{p}^{2}$ = .52, and interpretation, *F*(2,30) = 6.80, *p* = .004, $\eta_{p}^{2}$^ ^= .31, and the Object × Interpretation interaction was significant, *F*(2, 30) = 3.35, *p* = .049, $\eta_{p}^{2}$^ ^= .18. Error rates were overall comparable for simple objects (2.5%, 2.3%, and 3.5% for global, local, and mosaic shapes, respectively, *p*s > .12); for composite objects, by contrast, fewer errors were made with global (4.8%) than with local (7.7%) and mosaic (7.7%) interpretations (*p*s < .02). No other significant effects were obtained (*p*s > .28).

#### Reaction times

**Figure 3.**
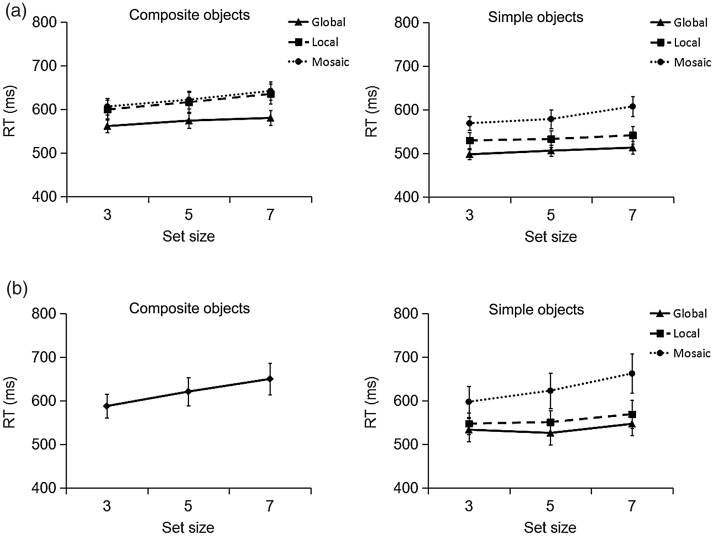
Mean search RTs in Experiments 1 (a) and 2 (b) for composite (left) and simple objects (right) on target-present trials, as a function of interpretation and set size. RT = reaction time.

Outliers (±2 *SD*s from the individual mean of each observer for each completion type) and error responses were excluded from the RT analysis (overall 4% of all trials). Figure 3(a) presents the mean correct RTs as a function of set size, separately for all possible interpretations of the composite (left panel) and simple objects (right panel).

A repeated-measures ANOVA with the factors object, interpretation, and set size revealed main effects of object, *F*(1, 15) = 82.27, *p* < .0001, $\eta_{p}^{2}$^ ^= .85, and interpretation, *F*(2, 30) = 19.37, *p* < .0001, $\eta_{p}^{2}$^ ^= .56. In addition, the Object × Interpretation interaction was significant, *F*(2, 30) = 11.41, *p* < .0001, $\eta_{p}^{2}$^ ^= .43. For simple objects, the RTs were slowest for the mosaic (585 ms), intermediate for the local (535 ms), and fastest for the global (506 ms; *p*s < .03) interpretation. For composite objects, by contrast, RTs were faster for global (572 ms) than for local (617 ms) and mosaic (623 ms; *p*s < .002) interpretations, without a difference between local and mosaic interpretations (*p = *.59). Note that the relatively fast RTs for the global interpretation in composite objects were nevertheless slower than the corresponding RTs for simple global objects, *t*(15) = 9.17, *p* < .0001. Finally, there was also a main effect of set size, *F*(2, 30) = 21.49, *p* < .0001, $\eta_{p}^{2}$^ ^= .59, revealing a 25-ms increase in RTs from the smallest to the largest set size (*p*s < .018). No other effects were significant (*p*s > .12).

### Discussion

The results of Experiment 1 demonstrate that simple-object search varied across the different interpretations of the cross-shaped target, revealing relatively efficient search (in terms of the mean RTs) with global followed by local and mosaic object types. This indicates that search varied as a function of the complexity of the objects’ shape, with more complex shapes reducing the search efficiency (see, e.g., Conci, Müller, & Elliott, 2007, 2009). In contrast, for composite objects, only search for the globally completed target afforded faster (and more accurate) responses compared to local- and mosaic-type objects (even though the perceptual input was identical in all cases). However, search for a global interpretation of the composite object was still less efficient than search for a simple global target, which potentially resulted from a perceptual difference between these two types of stimuli (e.g., due to the additional, occluding square in composite objects, see Figure 1(a)). Despite these perceptual differences, our results show that the context provided by trials presenting simple objects (in a given part of the experiment) engendered some form of “priming” that affected the interpretation of the concurrent composite objects (Chen et al., 2016, 2018; Plomp & Van Leeuwen, 2006). In line with previous findings in a working memory task (Chen et al., 2016, 2018), priming by the simple-object context led to an increased search efficiency for the globally completed (composite) objects—indicating that the completion of a symmetrical global object can facilitate search.

## Experiment 2

Experiment 2 was performed to further test the above hypothesis that the simple-object context primed search for (globally completed) composite objects—by examining whether completion would be evident in composite objects even when no consistent context is available. This was realized in Experiment 2 by presenting the various types of simple objects within the same experimental part—separated from another part that presented composite object trials. If the interpretation of a given composite object is determined mainly by the context of simple objects prevailing throughout a block of trials (as suggested by Experiment 1), then removing the informative context should eliminate any benefit of completion for the composite objects in Experiment 2.

### Method

Experiment 2 was essentially identical to Experiment 1. A new group of 16 right-handed volunteers (eight males, mean age = 27.06) was tested. All participants had normal or corrected-to-normal vision and were naive concerning the purpose of the experiment. Simple and composite objects were presented in two separate experimental parts. In the simple-object part, participants were asked to report the presence or absence of a cross-shaped target, which could be either the global, the local, or the mosaic “interpretation” of the cross (Figure 1(a), simple) among other simple object distractors of the same completion type for a given trial (Figure 1(b)–(d), simple). Thus, this part of the experiment presented one of the three possible target shapes, with the particular shape selected at random from one trial to the next. In the composite-object part, observers searched for the composite cross (Figure 1(a), composite) among other composite distractors (Figure 1(b)–(d), composite). Thus, for composite objects, no differentiation between global, local, and mosaic object types was possible; accordingly, the composite object trials were collapsed into a single condition. The experiment started with one block of 18 practice trials (presenting either simple or composite search displays), followed by five experimental blocks of 108 trials each within a given part, amounting to 1,116 trials in total (as in Experiment 1). Participants were presented with the simple- and composite-object parts in randomized order.

### Results

#### Errors

The mean error rate was 2.4%. A repeated-measures ANOVA with the factors object and set size revealed no significant effects (*p*s > .05). A subsequent ANOVA performed only on the simple objects also revealed no significant difference among the global, local, and mosaic interpretations (*p*s > .05).

#### Reaction times

Outliers and error responses were again excluded from the RT analysis (3% of all trials). Figure 3(b) presents the mean correct RTs as a function of set size, separately for the composite (left panel) and simple objects (right panel). A repeated-measures ANOVA, with the factors object and set size, revealed main effects of object, *F*(1, 15) = 5.29, *p* = .036, $\eta_{p}^{2}$^ ^= .26, and set size, *F*(2, 30) = 19.29, *p* < .0001, $\eta_{p}^{2}$^ ^= .56, and a significant interaction, *F*(2, 30) = 7.09, *p* = .003, $\eta_{p}^{2}$^ ^= .32. This effect pattern shows that search for the composite target was less efficient than simple object search (mean search slopes of 16 and 8 ms/item, respectively). Next, an ANOVA on the simple objects with the factors interpretation and set size again revealed both main effects—interpretation, *F*(2, 30) = 26.10, *p* < .0001, $\eta_{p}^{2}$^ ^= .64; set size, *F*(2, 30) = 18.64, *p* < .0001, $\eta_{p}^{2}$^ ^= .55—and their interaction, *F*(4, 60) = 3.54, *p* = .012, $\eta_{p}^{2}$^ ^= .19, to be significant. Search was less efficient for mosaic (16 ms/item) than for global (3 ms/item) and local (5 ms/item) simple object interpretations (*p*s < .02).

An additional analysis compared search for the composite object target to the various types of simple objects. This analysis revealed that composite object search was less efficient than simple global, *t*(15) = 3.42, *p* = .004, and simple local, *t*(15) = 4.32, *p* = .001, object search; but search for composite objects was comparable to search for simple mosaic objects, *t*(15) = .15, *p* = .89, indicating a relatively inefficient search for composite objects.

Further analyses compared the simple and composite object conditions between Experiments 1 and 2 (see Figure 3). First, for *composite objects*, the search slopes in Experiment 2 were comparable to the corresponding slopes for mosaic—9 ms/item; *t*(30) = 1.49, *p* = .15—and local—9 ms/item; *t*(30) = 1.43, *p* = .16—interpretations of composite objects in Experiment 1, but they were higher than for the composite, global object interpretation—5 ms/item; *t*(30) = 2.41, *p* = .02.

Second, for *simple objects*, a mixed ANOVA revealed main effects of interpretation, *F*(2, 60) = 49.13, *p* < .0001, $\eta_{p}^{2}$^ ^= .62, and set size, *F*(2, 60) = 30.89, *p* < .0001, $\eta_{p}^{2}$^ ^= .51, and an interaction between the two factors, *F*(4, 120) = 5.25, *p* = .001, $\eta_{p}^{2}$^ ^= .15. Search was less efficient for mosaic (13 ms/item) than for global (4 ms/item) and local (4 ms/item) interpretations, but without revealing differences between the two experiments (*p*s > .2). In other words, searching for the various types of simple objects was overall comparable across the two experiments.

### Discussion

The results of Experiment 2 closely replicated the pattern of results for simple objects in Experiment 1, showing comparable variations in search across global, local, and mosaic object interpretations. For composite objects, search was found to be comparable to simple mosaic objects (but less efficient than for local or global simple objects). Moreover, the search slopes for composite objects in Experiment 2 were also comparable to local and mosaic composite object interpretations in Experiment 1 but higher than the global composite interpretation. This pattern suggests that without simple-object priming, no completion occurs and search remains inefficient as a result (with search performance essentially being comparable to the uncompleted mosaic condition). The combined results of Experiments 1 and 2 thus indicate that a given consistent context can expedite search for occluded objects.

## General Discussion

The present results show that visual search for composite objects can be facilitated by amodal completion. Experiment 1 revealed faster (and more accurate) search when a composite object was interpreted as a globally completed whole, while search in physically identical displays was comparably more inefficient in blocks associated with an interpretation in terms of a locally completed whole or for an uncompleted mosaic object. Importantly, this effect was obtained only when the simple-object context supported a coherent interpretation of the composite shapes (Experiment 1); without any such informative context, searching displays of composite objects was comparably inefficient to uncompleted mosaic objects (Experiment 2).

A potential explanation to account for the advantage of global object completions appeals to the internal representation used to guide search, that is, some form of “attentional template”. Such templates are thought to hold target representations stored in visual working memory, top-down biasing search to any objects in the visual array that match the template (Desimone & Duncan, 1995; Humphreys & Müller, 1993). From this perspective, our results would indicate that, given a predictive simple-object context, a globally completed template representation is set up preferentially and this regular, symmetric specification of the target then in turn expedites performance by directly guiding focal attention to the (partially matching) target object—which, as a result of the top-down projection, is then actually interpreted or “perceived” as globally completed. This is consistent with previous findings that have established a link of object completion to working memory processes (Chen et al., 2016, 2018) and suggests that some imagery process completes the missing, occluded information, thereby generating an effective, perceptually simple template (a global “Gestalt”) that in turn expedites target detection. Although a symmetric global target representation facilitated search, representations of mosaic-type or locally completed targets were found not to be comparably efficient in providing search guidance—likely because they are more difficult to construct and maintain as a template in visual working memory.

It should also be noted that, in Experiment 2 (without consistent context), search for composite objects was comparable in efficiency to search for the simple-object mosaic condition. This suggests that completion is not automatic but comes into play only when the context consistently enforces a given interpretation of the composite object (Chen et al., 2016, 2018; Rauschenberger et al., 2004). Contextual knowledge or prior expectations, rather than bottom-up grouping, might therefore determine the interpretation of an occluded object (see also Conci & Müller, 2014)—the implication being that amodal completion, in fact, reflects a cognitive, rather than a perceptual, mechanism.

One potential limitation of this study is that an effect of completion was demonstrated with a rather small stimulus set, namely, by comparing search performance for the various types of the cross-shaped target (Figure 1(a)). That is, the observed effects might be due to particularities of this shape configuration—where the global object interpretation (of a symmetric cross) is more familiar than the corresponding local and mosaic variants. Of note, however, we used the very same stimulus set in a previous study that employed a change detection task (Chen et al., 2016). In these experiments, the target item (i.e., the item that exhibited a to-be-detected change) varied from trial to trial, and the various targets were also variable in terms of their familiarity. Nevertheless, a robust advantage for the global interpretation was observed, suggesting that the current results based on a single target shape do generalize to other shapes. Moreover, a study by Shen and Reingold (2001) showed that more familiar search stimuli might even lead to a reduction of search performance (relative to an unfamiliar stimulus set). This again argues that the observed benefit for the global interpretation is not simply attributable to object familiarity.

In summary, the present findings indicate that amodal completion operations may enhance search by providing, in particular, a symmetric global shape representation (template) to guide attention effectively to the target. However, global object completion does not rely on automatic integration processes but does instead critically depend on the search environment providing a coherent interpretational context—in line with the notion of an experience-dependent template that represents a completed search image of the occluded (target) object.
